# Protein Kinase C Phosphorylates the System N Glutamine Transporter SN1 (Slc38a3) and Regulates Its Membrane Trafficking and Degradation

**DOI:** 10.3389/fendo.2013.00138

**Published:** 2013-10-02

**Authors:** Lise Sofie H. Nissen-Meyer, Farrukh Abbas Chaudhry

**Affiliations:** ^1^The Biotechnology Centre, University of Oslo, Oslo, Norway; ^2^The Institute of Basic Medical Sciences, University of Oslo, Oslo, Norway

**Keywords:** SN1, Slc38, glutamine, glutamate, PKC, GABA, neurotransmitter replenishment, transporter

## Abstract

The system N transporter SN1 (also known as SNAT3) is enriched on perisynaptic astroglial cell membranes. SN1 mediates electroneutral and bidirectional glutamine transport, and regulates the intracellular as well as the extracellular concentrations of glutamine. We hypothesize that SN1 participates in the glutamate/γ-aminobutyric acid (GABA)-glutamine cycle and regulates the amount of glutamine supplied to the neurons for replenishment of the neurotransmitter pools of glutamate and GABA. We also hypothesize that its activity on the plasma membrane is regulated by protein kinase C (PKC)-mediated phosphorylation and that SN1 activity has an impact on synaptic plasticity. This review discusses reports on the regulation of SN1 by PKC and presents a consolidated model for regulation and degradation of SN1 and the subsequent functional implications. As SN1 function is likely also regulated by PKC-mediated phosphorylation in peripheral organs, the same mechanisms may, thus, have impact on e.g., pH regulation in the kidney, urea formation in the liver, and insulin secretion in the pancreas.

## Introduction

Synaptic transmission at a chemical synapse is essential to many neuronal functions such as cognition, learning, and memory. It is based on exocytotic release of a neurotransmitter, its diffusion through the synaptic cleft and activation of specific receptors on the surface of the target cell. Glutamate is the major fast excitatory neurotransmitter in the central nervous system (CNS) undergirding the function of a wide range of synapses, while γ-aminobutyric acid (GABA) and glycine are the primary inhibitory neurotransmitters involved, among others, in synchronization of the principal neurons. In addition, monoamines, acetylcholine, neuropeptides, and other molecules sustain neuronal signaling at specific synapses [for review see Ref. (1)].

**Figure 1:**
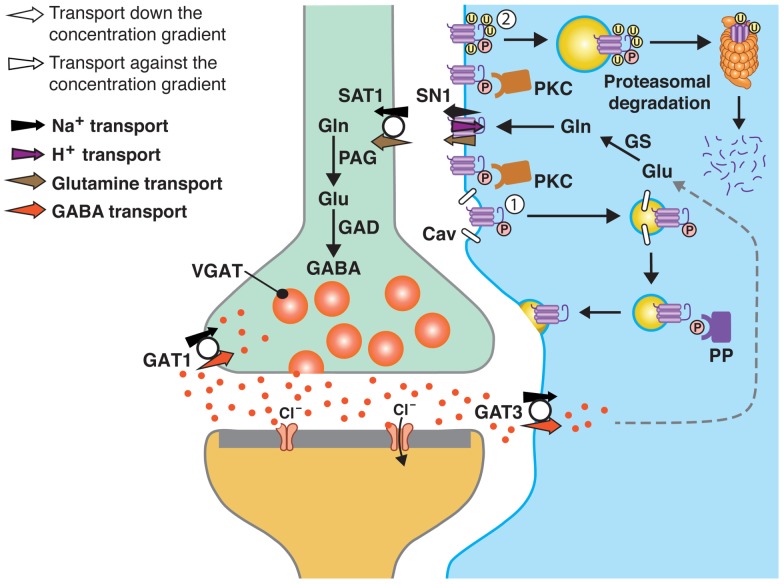
**A model of SN1 membrane trafficking based on consolidated data on the regulation of SN1 activity by protein kinase C (PKC)**. The cartoon depicts a GABAergic synapse in adult rat brain where GABA is released exocytotically and acts upon specific post-synaptic receptors. The signal is terminated by removal of GABA from the synaptic cleft by transport of GABA back into the nerve terminal by the plasma membrane GABA transporter (GAT) 1. A substantial amount of GABA in the synaptic cleft is also transported into perisynaptic astroglial processes by GAT3 (and GAT1) and converted to glutamate, and then to glutamine catalyzed by glutamine synthetase (GS). Glutamine may then be released from the astroglial cells by the electroneutral and bidirectional system N transporter 1, SN1, and may subsequently be accumulated inside GABAergic neurons by the system A transporter SAT1. Here, glutamine is metabolized to yield glutamate and then GABA by the action of phosphate-activated glutaminase (PAG) and glutamic acid decarboxylase (GAD), respectively. Finally, GABA is translocated into synaptic vesicles by the vesicular GABA transporter (VGAT), wherefrom it is ready to be exocytotically released. Astroglial PKC may be activated upon stimulation of specific receptors on the astroglial membranes or e.g., by excessive amounts of Mn^2+^. PKCα and PKCγ (and PKCδ) can phosphorylate SN1 on a serine at the 52 position. This results in caveolin (Cav)-dependent internalization of SN1 from the plasma membrane (1). The internalized SN1 may be relocated to the plasma membrane upon dephosphorylation by protein phosphatases (PP). PKC-mediated phosphorylation of SN1 also increases ubiquitination of SN1 (2). This may also internalize the protein into intracellular compartments and target it to the proteasomal degradation pathway. Similar regulation of SN1 activity also takes place at glutamatergic synapses. The same mechanisms are likely to be involved in the regulation of SN1 in hepatocytes, renal tubule cells, and the pancreatic B-cells.

Sustained neurotransmission is dependent on replenishment of the neurotransmitters and efficient termination of the signal to reduce signal-to-noise ratio. In the case of the monoamines and acetylcholine, the neurotransmitters or choline (end-product of acetylcholine hydrolysis by acetylcholinesterase) are removed from the synaptic cleft by specific transporters on the nerve terminal membranes which also allow for their reuse in synaptic transmission (1). In contrast, the GABA transporter 3 (GAT3; and partially GAT1) and the major glutamate transporters (GLAST/EAAT1 and GLT-1/EAAT2) reside on surrounding astroglial cells, and a drain of these transmitters to the astroglial cells has been demonstrated (Figure 1) (2, 3). With the characterization of the members of the Slc38 family of amino acid transporters, showing that they may work in concert to shuttle glutamine from astroglial cells to neurons, the theory on a glutamate/GABA-glutamine cycle has been revitalized. In particular, the astroglial SN1, which releases glutamine, seems to be a major component of this cycle as it regulates extracellular concentrations of glutamine and shows dynamic membrane trafficking (4–6). In this review, we will discuss and consolidate recent data on the protein kinase C (PKC)-mediated regulation of SN1 and its functional implications.

## The Glutamate/GABA-Glutamine Cycle and Its Contribution to the Replenishment of the Neurotransmitters Glutamate and GABA

The considerable amount of transmitter steadily released from neuronal synapses demands a dependable mechanism for replenishment. The neurotransmitters glutamate and GABA cannot be generated from the tricarboxylic acid (TCA) cycle intermediates because neurons lack the ability for anaplerosis due to lack of pyruvate carboxylase (7). Indeed, glucose alone is insufficient to sustain neurotransmission in brain slices (8). Shuttling of monocarboxylates and TCA cycle intermediates from astroglial cells to neurons and contribution to the formation of the neurotransmitters has also been suggested (7, 9), but it remains to be demonstrated that they can undergird neurotransmitter synthesis and synaptic transmission. The prevailing hypothesis is therefore that the fast neurotransmitters shuttle through perisynaptic astroglial cells in order to sustain neurotransmission. According to this glutamate/GABA-glutamine cycle, glutamate and GABA are sequestered into astroglial cells and converted to glutamine. Astroglial cells then supply neurons with glutamine to fuel formation of glutamate and GABA.

There are several compelling findings supporting existence of the glutamate/GABA-glutamine cycle and that astroglial-derived glutamine is the primary precursor for the neurotransmitters glutamate and GABA (Figure 1). Astroglial cells ensheath synapses, furnish neurons with metabolic precursors and optimize conditions for neuronal function and signaling. They harbor GLAST, GLT-1, and GAT3 and quickly remove the neurotransmitters from the synaptic cleft and away from their receptors, by binding the neurotransmitters and subsequently transporting them into astroglial cells [(10–12), review (3)]. The sequestered glutamate and GABA are readily metabolized to glutamine by glutamine synthetase, which is enriched in astroglial cells and unique in being capable of synthesizing glutamine in the human body (13, 14). Glutamine transported into nerve terminals is catabolized by the phosphate-activated glutaminase (PAG), which is pronounced in nerve terminals, to resynthesize glutamate and GABA [for review see Ref. (15)]. Finally, the newly synthesized glutamate and GABA is accumulated inside synaptic vesicles by vesicular transporters prior to their exocytotic release (16, 17). Glutamine as a precursor for the neurotransmitters glutamate and GABA has been demonstrated beyond any doubts (7, 8, 18–22). However, how glutamine is shuttled from astroglial cells, where it is synthesized, into neurons for its utilization has been enigmatic.

## The System N and System A Transporters Sustain Astroglial-to-Neuron Transport of Glutamine

The break-through in our understanding of the intercellular transport of glutamine and the glutamate/GABA-glutamine cycle was established by characterization of an orphan transporter homologous to the vesicular GABA transporter (VGAT): SN1 – a 504 amino acids long transporter with 11 putative transmembrane domains and a long intracellular N-terminal – transports glutamine, asparagine, and histidine consistent with the biochemically described system N activity (4). SN1 transport is coupled to Na^+^ transport in symport and H^+^ transport in antiport. Consequently, SN1 activity is associated with intracellular pH changes. The stoichiometric coupling of SN1 to Na^+^ and H^+^ running in opposite directions, makes the overall transport electroneutral and allows SN1 to work bidirectionally (4, 23–25). In addition to the coupled movement of Na^+^ and H^+^ ions, cations also penetrate SN1 in an uncoupled manner and enable SN1 to readily work in the release mode at physiological conditions. In the CNS, SN1 is localized on astroglial processes ensheathing synapses (5, 23, 26). During synaptic transmission and the subsequent depolarization of astroglial cells, ion and glutamine concentration gradients change and favor the release mode of SN1 (27). SN1 is therefore able to furnish nerve terminals with glutamine for neurotransmitter synthesis.

Interestingly, molecular identification of SN1 revealed a family of amino acid transporters (Slc38) including SN2 and the unidirectional system A transporters SAT1 and SAT2 [for review see Ref. (27, 28)]. The isoform-specific characteristics of these transporters together with their complementary localization enable these transporters to sustain intercellular transport of glutamine (Figure 1). SAT1 is pronounced in GABAergic neurons in the CNS, targeted to growth cones in developing neurons and to the same cellular compartments as VGAT in the mature intact neurons, indicating a role in glutamine uptake for GABA formation (29–31). In contrast, SAT2 is enriched in the somatodendritic compartments of glutamatergic neurons throughout the CNS and accumulates high levels of glutamine (32, 33). Upon stimulation of these neurons, glutamine is metabolized to generate glutamate which is released from their dendrites. Indeed, a pharmacologic disruption of SAT2 abolishes retrograde signaling (33). Finally, the system N transporter SN2 is exclusively expressed on astroglial cell membranes and mediates electroneutral and bidirectional transport of several neutral amino acids (34, 35). SN2 participates in astroglial release of glutamine for neurotransmitter generation but adds on by releasing glycine for co-activation of NMDA receptors (35).

## A Diverse Range of Mechanisms are Involved in the Regulation of the System A and System N Transporters

As the Slc38 family of amino acid transporters sustains astroglial-to-neuron transport of glutamine, regulation of the system A and system N transporters may have impact on neurotransmitter replenishment and synaptic plasticity. A better understanding of molecular mechanisms involved in their function and regulation may reveal novel (patho-)functional roles of these transporters and the glutamate/GABA-glutamine cycle and unveil novel therapeutic targets. Eukaryotic cells have an entire range of possible regulatory mechanisms to regulate the activity and expression of their proteins. In principle, all steps of production, maturation, trafficking, and degradation of a cellular protein can be regulated to control its expression levels, in addition to all types of direct or indirect influence of protein activity mediated by interaction with molecules ranging from protons to macromolecular protein complexes. The glutamine transporters are no exception to this rule. Classical biochemical experiments early demonstrated adaptive, hormonal, and osmotic regulation of system A and N activities as measured by functional transport assays (36–38). A nutrition signaling cascade that includes activation of phosphatidylinositol 3-kinase (PI3K) and mammalian target of rapamycin (mTOR) has been shown to be important for upregulation of system A (39, 40). For SAT2, an amino acid response element regulating the promoter activity has been demonstrated (41).

The system N activity and SN1 is regulated at the transcriptional and translational levels (37, 42). Sophisticated regulation of SN1 by interacting proteins and ions has also been demonstrated. SN1 is e.g., targeted by the ubiquitin ligase Nedd4-2 (neural precursor cell expressed, developmentally down-regulated 4-2) which down-regulates SN1 activity in *Xenopus laevis* (*X. laevis*) oocytes (43). Insulin regulates expression of SN1 through the PI3K-mTor signaling cascade (44).

Protons regulate SN1 activity by competing with Na^+^ at the sodium binding site of SN1 as shown by increasing *K*_m_ for Li^+^ (a substitute for Na^+^) with little change in *V*_max_ upon reducing extracellular pH (23). As Na^+^ binding to the Slc38 family is a prerequisite for the binding of the amino acid prior to its translocation, extracellular pH changes have profound effect on SN1 activity (23, 24). Protons also regulate SN1 at the mRNA level. SN1 expression at normal conditions is restricted to the S3 segment of the proximal tubules of the kidneys. During chronic metabolic acidosis (CMA), SN1 is induced also in the S1–S2 segments of renal epithelium, thereby increasing glutamine metabolism and generation of bicarbonate to counteract acidosis (6). Induction of CMA in rats results in upregulation of SN1 by about 10-fold at the mRNA level and more than 5-fold at the protein level (6, 42, 45). A pH responsive element in the 3′ untranslated region (3′-UTR) of SN1 mRNA allows binding of specific proteins to the mRNA at low pH and thereby stabilizes the mRNA. As a result, SN1 expression is induced in the entire S1–S3 segments of the kidney (6).

## PKC-Mediated Phosphorylation is Central for Regulating Membrane Trafficking of Plasma Membrane Neurotransmitter Transporters

For proteins transporting neuroactive compounds, such as dopamine, serotonin (SERT), GABA, and glutamate, membrane trafficking regulated by phosphorylation/dephosphorylation events has been shown as a common denominator. Nedd4-2/serum and glucocorticoid inducible kinases 1 and 3 (SGK1 and 3) and protein kinase B (PKB) regulation have been described for the glutamate transporters EAAT1, 2, and 5 (46). However, the regulation by PKC stands out as a major mechanism. PKC isoforms and their numerous substrates regulate a variety of membrane proteins and in particular transporters [reviewed by (47, 48)]. In a comprehensive review, transporters for GABA (GAT1), SERT, dopamine (DAT1), and glutamate (EAAC1) were all shown to be regulated by PKC phosphorylation (48). For the three first transporters, PKC phosphorylation mediates internalization of the transporters, whereas dephosphorylation by Protein Phosphatase 2A (PP2A) or tyrosine phosphorylation mediates trafficking back into the cell membrane. For EAAC1, PKC seems to increase the surface expression together with PI3-kinase, whereas PP2A dephosphorylation elicits the internalization. Also the glycine transporter is being regulated by PKC (49–51). As PKC is ubiquitously expressed but strictly compartmentalized, its subcellular activation is differentially executed through a myriad of signal pathways, accounting for a large and diverse part of total phosphorylation phenomena in all cell types. Interestingly, PKC-signaling has been shown important for CNS processes like neuronal development, excitability, plasticity, and aging (52–54). Given the central regulatory role of PKC it is not surprising that the idea of PKC-regulation also of glutamine transporters was conceived by several different groups independently.

## Evidence of PKC-Mediated Phosphorylation of SN1

Protein kinase C has recently been shown to regulate SN1 under physiological conditions by two groups (Figure 1), however, there is a difference in the reported mechanisms involved (55, 56). Balkrishna and co-workers report that treatment of *X. laevis* oocytes expressing rat SN1 with the phorbol ester phorbol 12-myristate 13-acetate (PMA) results in a rapid down-regulation of glutamine uptake in <20 min. As this PMA-induced reduction in glutamine uptake is prevented by the specific PKC-inhibitor bisindolylmaleimide (Bis) and could not be induced by an inactive form of PMA (4-α-PMA), the investigators conclude that PKC activation is involved. The specificity of the PKC action on SN1 was supported by lack of PMA-induced changes in the monocarboxylate transporter 1 (MCT1) activity in the same oocytes. The authors identify seven putative phosphorylation sites in the SN1 sequence, however, single or combined mutations of these sites have no impact on the PMA-induced down-regulation of SN1 activity. In an effort to further identify presence of particular regions or motifs on SN1 responsible for the observed PKC effect, the SN1 cytosolic N-terminus was replaced with the N-terminal part of the homologous SAT1 protein. Treatment of the SAT1-SN1 hybrid with PMA still resulted in down-regulation of SN1, a result interpreted as proof that the targeting region is not localized in the SN1 N-terminus. Based on all these data it is concluded that SN1 is not directly phosphorylated by PKC; rather, the down-regulation of SN1 is mediated by some interaction with regulatory proteins endogenous to oocytes (55).

The authors also generate a fluorescently labeled construct of SN1, EGFP-SN1, and show that PKC-mediated down-regulation of glutamine uptake is caused by internalization of the fusion protein from the plasma membrane. The retrieval of SN1 from the plasma membrane is controlled by caveolin but remains dynamin-independent. Lastly, glutamine transport is challenged in the hepatocyte-derived HepG2 cells and cultured rat astrocytes; both hepatocytes and astrocytes have endogenous SN1 expression (26). PMA-treatment reduces glutamine uptake in cultured HepG2 cells but not in their cultured rat astrocytes, and this discrepancy is explained by differences in cell-specific regulatory mechanisms.

In the report by Nissen-Meyer and co-workers (56), we also detect a comparable time-dependent down-regulation of SN1 protein in the plasma membrane following PMA-treatment of mammalian cells stably transfected with SN1, and sequestration of the protein into intracellular reservoirs. The down-regulation is inhibited in the presence of Bis I, supporting mediation by PKC activation. However, we demonstrate PKC-mediated phosphorylation of SN1 in three different ways: first, direct phosphorylation of SN1 *in vitro* using a GST-fusion protein containing the N-terminal of SN1 and recombinant PKCα and PKCγ (56). Further, using site-directed mutagenesis to create unphosphorylatable SN1 mutants, we transfected cultured COS7 and PS120 cells with these mutant plasmids. Cells were then metabolically labeled with ^32^P-orthophosphate, stimulated with PMA and following immunoprecipitation and 2D-phosphopeptide mapping, we demonstrated that PKC-dependent phosphorylation in living cells was abolished selectively when a single serine residue was mutated (S52A) in the N-terminal of rat SN1, implicating this as the primary phosphorylation site.

Second, characterization of wild type and mutant SN1 in *X. laevis* oocytes electrophysiologically further corroborated our data on direct phosphorylation of SN1 by PKC. PMA-stimulation results in reduced SN1 activity as shown by abolished glutamine-induced inward currents. However, such reduction in the magnitude of the glutamine-induced inward currents perish when PKC is inhibited by Bis I. The unphosphorylatable S52A mutant resisted down-regulation in the presence of PMA, implicating that PKC isoforms phosphorylate SN1 at the S52.

Third, we also show direct phosphorylation of SN1 in cultured rat astroglial cells. By using specific affinity-purified antibodies selectively recognizing SN1 phosphorylated at the S52, we demonstrated that PKC stimulation results in increasing Bis I-sensitive phosphorylation of SN1 and that phosphorylated SN1 accumulates in intracellular compartments consistent with internalization of the protein. Such internalization of SN1 upon PKC-mediated phosphorylation is also supported by the fact that PKC activation significantly reduces *V*_max_ of the glutamine-induced currents in *X. laevis* oocytes but has no effect on the *K*_m_. Finally, our biochemical analyses suggest that SN1 may dynamically be recruited from these compartments upon dephosphorylation, however, prolonged activation of SN1 by PKC results in its degradation.

Interestingly, Sidoryk-Wegrzynowicz and co-workers recently also presented evidence that PKC is involved in the down-regulation of SN1 (57): Mn^2+^ exposure upregulates the activity of both PKCα and PKCδ in cultured astrocytes. Both enzymes were activated by phosphorylation and PKCδ was in addition activated by caspase 3-dependent proteolysis. In their experiments, PMA-stimulation for 4 h significantly down-regulates system N-mediated glutamine uptake in cultured astrocytes, an effect which was inhibited by addition of the PKC-inhibitor Bis II. In harmony with our work, they show that PMA reduces the SN1-content of biotinylated surface membranes long before 4 h. Although they did not succeed in co-immunoprecipitating SN1 with PKCα, they did show that SN1 co-immunoprecipitates together with PKCδ at 0 and 2 h, but not at the later times investigated. Thus, these experiments also lend support to PKC being an important regulator of SN1 protein cell surface expression under physiological conditions, albeit not demonstrating direct phosphorylation of SN1. Thus, there are compelling evidence and some indications that SN1 is directly phosphorylated by PKCα, PKCγ, and PKCδ and that this is followed by caveolin-dependent internalization of SN1.

## Is PKC Involved in the Degradation of SN1?

As shown above, PKC phosphorylates SN1 and regulates SN1 activity on cell membranes and thereby adjusts the transmembrane glutamine transport to comply with different (patho-)physiological demands for the neurotransmitter precursor. However, prolonged activation of PKC results in degradation of SN1 (Figure 1) (56, 57). SN1 interacts with Nedd4-2 when co-expressed in *X. laevis* oocytes and in astrocytes (43, 57). Moreover, stimulation of primary astrocytes with Mn^2+^ induces ubiquitin/proteasome-mediated degradation of SN1 via the Nedd4-2/SGK1 signaling pathway, thus providing a partial explanation for Mn^2+^-induced neurotoxicity (58). Consequently, SN1-mediated transport increases when SN1 is co-expressed with the SGK1 and 3, and PKB (43). Thus, this pathway could represent a link to PKC-regulation since phosphorylation by PKC frequently is a way to tag proteins for ubiquitination and further lysosomal or proteasomal degradation (Figure 1) (59, 60).

## What is the Functional Significance of SN1 Regulation for the Glutamate/GABA-Glutamine Cycle?

The glutamate transporters GLAST and GLT-1 and the GABA transporters GAT1 and GAT3 are enriched on the cell membranes of perisynaptic astroglial processes, capturing the exocytotically released neurotransmitters and translocating them into astroglial cells (10, 11). SN1 is also targeted to the same small glial processes (5, 26). Activation of the glutamate and GABA transporters will ensure that the local intracellular Na^+^-concentration can be increased to a level where it can drive the SN1-mediated glutamine transport out of the cell (61), and as long as glutamine synthetase is present, glutamate and GABA imported will be transformed to glutamine for export. Indeed, glutamate stimulates efflux of glutamine from astroglial cells (62).

Uwechue and co-workers have provided compelling evidence that glutamate evokes release of glutamine through a system N like activity in astrocytes juxtaposed to the glutamatergic calyx of Held synapse in the rat medial nucleus of the trapezoid body (MNTB). Subsequently, such glutamine release is sensed by MNTB principal neurons which express system A transporters consistent with an intact glutamate/GABA-glutamine cycle (63, 64). Similarly, studies on cultured Bergmann glia cells show that activation of glutamate transporters by d-aspartate results in release of glutamine (65). Altogether, these data strongly suggest functional coupling between the glutamate and glutamine transporters and existence of a glutamate/GABA-glutamine cycle. Taken together with the dynamic regulation of the membrane trafficking of SN1 activity (55–57) this suggests that SN1 may be one of the key regulators of neuronal supply of glutamine and thus the glutamate/GABA-glutamine cycle (Figure 1). In addition, inhibition of SN1 activity may stimulate targeting of glutamate, GABA, and glutamine for oxidation or increase trans-astrocytic glutamine fluxes with impact on surrounding regions (66).

## PKC-Mediated Phosphorylation may Regulate a Wide Range of Functions in Peripheral Organs

SN1 also sustains pivotal functions in peripheral organs. In the kidney, SN1 is localized on the basolateral membranes of the S3 segment of proximal tubules and is essential for glutamine metabolism. During CMA, K^+^-deprivation, and/or high protein intake, the total levels of SN1 increase significantly in the kidney and SN1 is also induced in the S1–S2 segments of the nephron (6, 45, 67). In the endocrine pancreas, we have shown complementary expression of SN1 and SAT2 and suggested that they work in concert to regulate a local glutamate-glutamine cycle and secretion of insulin (68, 69). The liver has one of the highest cellular concentrations of SN1 and is suggested to mediate glutamine influx for urea formation in periportal hepatocytes and glutamine efflux from the perivenous hepatocytes for transport of glutamine to other peripheral organs for cellular metabolism (26, 27). Accordingly, SN1 expression in the liver is regulated during starvation and insulin secretion (44). Thus, SN1 is essential in several physiological processes and may be differentially regulated in different organs to optimize a wide range of functions. As PKC isoforms are ubiquitously expressed throughout the body, but differentially in different cellular and subcellular compartments, isoform-specific PKC-mediated phosphorylation of SN1 may have a range of physiological and pathological roles.

## Conclusion

Consolidated data from several papers on the regulation of SN1 (55–57) show that PMA-induced and Bis I-inhibitable retrieval of SN1 occurs in several mammalian cell types, including primary rat astrocytes, and in *X. laevis* oocytes (Figure 1). Such membrane trafficking is governed by specific phosphorylation of SN1 at S52, selectively by PKCα and PKCγ. Prolonged PMA-stimulation results in internalization by a caveolin-dependent and dynamin-independent mechanism and to degradation of SN1 through the Nedd4-2/ubiquitination pathway. PKC-mediated regulation of SN1 may, thus, be a key step in the regulation of the glutamate/GABA-glutamine cycle in the CNS and a wide range of pathophysiological processes in peripheral organs. Further studies are required for a better understanding of molecular mechanisms governing regulation of SN1 activity on the plasma membrane and its membrane trafficking and such studies may reveal novel mechanistic insight into a variety of physiological processes and to discovery of novel therapeutic targets.

## Conflict of Interest Statement

The authors declare that the research was conducted in the absence of any commercial or financial relationships that could be construed as a potential conflict of interest.
