# Corrigendum: Protein Kinase C Phosphorylates the System N Glutamine Transporter SN1 (Slc38a3) and Regulates Its Membrane Trafficking and Degradation

**DOI:** 10.3389/fendo.2017.00190

**Published:** 2017-08-17

**Authors:** Lise Sofie H. Nissen-Meyer, Farrukh Abbas Chaudhry

**Affiliations:** ^1^The Biotechnology Centre, University of Oslo, Oslo, Norway; ^2^The Institute of Basic Medical Sciences, University of Oslo, Oslo, Norway

**Keywords:** SN1, Slc38, glutamine, glutamate, PKC, GABA, neurotransmitter replenishment, transporter

In the original article, there was a mistake in Figure 1 as published. The figure has two white arrows (in the left upper corner) which are labeled opposite of what they should be. The corrected Figure 1 appears below. The authors apologize for this error and state that this does not change the scientific conclusions of the article in any way.

**Figure 1:**
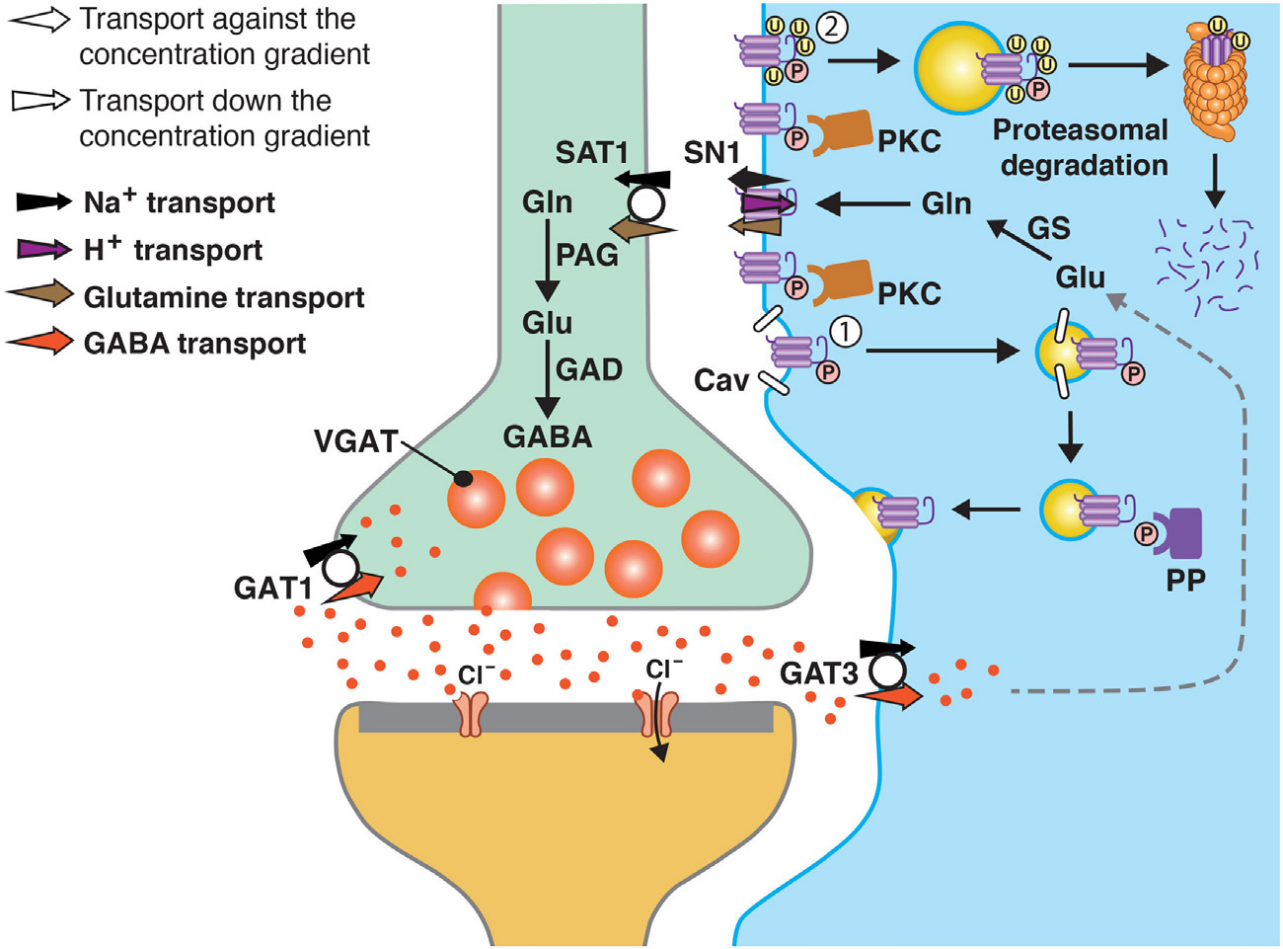
A model of SN1 membrane trafficking based on consolidated data on the regulation of SN1 activity by protein kinase C (PKC). The cartoon depicts a GABAergic synapse in adult rat brain where GABA is released exocytotically and acts upon specific post-synaptic receptors. The signal is terminated by removal of GABA from the synaptic cleft by transport of GABA back into the nerve terminal by the plasma membrane GABA transporter (GAT) 1. A substantial amount of GABA in the synaptic cleft is also transported into perisynaptic astroglial processes by GAT3 (and GAT1) and converted to glutamate, and then to glutamine catalyzed by glutamine synthetase (GS). Glutamine may then be released from the astroglial cells by the electroneutral and bidirectional system N transporter 1, SN1, and may subsequently be accumulated inside GABAergic neurons by the system A transporter SAT1. Here, glutamine is metabolized to yield glutamate and then GABA by the action of phosphate-activated glutaminase (PAG) and glutamic acid decarboxylase (GAD), respectively. Finally, GABA is translocated into synaptic vesicles by the vesicular GABA transporter (VGAT), wherefrom it is ready to be exocytotically released. Astroglial PKC may be activated upon stimulation of specific receptors on the astroglial membranes or e.g., by excessive amounts of Mn2C. PKCa and PKCg (and PKCd) can phosphorylate SN1 on a serine at the 52 position. This results in caveolin (Cav)-dependent internalization of SN1 from the plasma membrane (1). The internalized SN1 may be relocated to the plasma membrane upon dephosphorylation by protein phosphatases (PP). PKC-mediated phosphorylation of SN1 also increases ubiquitination of SN1 (2). This may also internalize the protein into intracellular compartments and target it to the proteasomal degradation pathway. Similar regulation of SN1 activity also takes place at glutamatergic synapses. The same mechanisms are likely to be involved in the regulation of SN1 in hepatocytes, renal tubule cells, and the pancreatic B-cells.

## Conflict of Interest Statement

The authors declare that the research was conducted in the absence of any commercial or financial relationships that could be construed as a potential conflict of interest.

